# Case report: Atomoxetine improves chronic pain with comorbid post-traumatic stress disorder and attention deficit hyperactivity disorder

**DOI:** 10.3389/fpsyt.2023.1221694

**Published:** 2023-08-07

**Authors:** Satoshi Kasahara, Miwako Takahashi, Taito Morita, Ko Matsudaira, Naoko Sato, Toshimitsu Momose, Shin-Ichi Niwa, Kanji Uchida

**Affiliations:** ^1^Department of Anesthesiology and Pain Relief Center, The University of Tokyo Hospital, Tokyo, Japan; ^2^Department of Pain Medicine, Fukushima Medical University School of Medicine, Fukushima, Japan; ^3^Institute for Quantum Medical Science, National Institutes for Quantum Science and Technology (QST), Chiba, Japan; ^4^Nursing Department, The University of Tokyo Hospital, Tokyo, Japan; ^5^Institute of Engineering Innovation, School of Engineering, The University of Tokyo, Tokyo, Japan; ^6^Department of Psychiatry, Aizu Medical Center, Fukushima Medical University, Fukushima, Japan

**Keywords:** atomoxetine, attention deficit hyperactivity disorder, cerebral blood flow, chronic pain, post-traumatic stress disorder, single-photon emission computed tomography

## Abstract

**Background:**

It is known that patients reporting chronic pain often experience trauma or post-traumatic stress disorder (PTSD) and tend to be more difficult to treat. Attention deficit hyperactivity disorder (ADHD), a neurodevelopmental disorder, is frequently associated with chronic pain. Furthermore, patients diagnosed with ADHD are more likely to encounter trauma and develop PTSD because of their inattentive and impulsive tendencies. There are reports stating that atomoxetine (ATX), a selective noradrenaline reuptake inhibitor for ADHD, is effective in patients diagnosed with PTSD and ADHD. However, there have been no reports on cases of comorbid PTSD and ADHD with chronic pain, and ATX’s potential in improving chronic pain coexisting PTSD. Furthermore, no reports have evaluated patient cerebral blood flow in conjunction with the course of treatment with ATX for chronic pain.

**Case report:**

In this study, we reported a case where ATX improved chronic pain with PTSD and improved cerebral blood flow. The patient was a 56-year-old woman exhibiting chronic pain with PTSD, resulting from 6 years of severe domestic violence from her common-law husband. She had no history of ADHD diagnosis, but through aggressive screening, comorbid ADHD was diagnosed. When treated with ATX, there were significant improvements in her pain, quality of life, anxiety, depression, catastrophic thoughts, and cerebral blood flow. As a result, she could resume work after 11 years.

**Conclusion:**

The study showed that chronic pain with PTSD may be comorbid with ADHD. Moreover, we found that ATX can improve chronic pain with PTSD and cerebral blood flow. Aggressive screening of ADHD is important because once the diagnosis of comorbidity is confirmed, an ideal ADHD treatment can be selected. Therefore, based on the results of this study, ATX may be a candidate for treatment for cases of chronic pain with PTSD and ADHD.

## 1. Introduction

Patients experiencing chronic pain are known to have encountered traumatic experiences and/or have developed post-traumatic stress disorder (PTSD). The treatment of such patients tends to be difficult because they usually exhibit severe pain, PTSD symptoms, anxiety, depression, functional impairment, and greater opioid use (1). Attention deficit hyperactivity disorder (ADHD), a neurodevelopmental disorder, is frequently associated with chronic pain, and the two are closely related (2–8). Furthermore, ADHD increases the chances of developing PTSD after experiencing a traumatic event because the individual exhibits inattentive and impulsive tendencies (9).

Of note, patients diagnosed with PTSD and ADHD are more likely to show cognitive dysfunctions in attention, working memory, executive function, and impulse control (10, 11), and present with functional somatic syndromes (9). The publication “The Body Keeps the Score,” reported that almost all of the 12 children who had suffered domestic abuse and visited the children’s clinic at the Massachusetts Mental Health Center had a previous diagnosis of ADHD (12). A meta-analysis between ADHD and PTSD showed that an ADHD diagnosis was associated with a four-fold increase in the risk of developing PTSD. Conversely, encountering traumatic experiences culminating in the development of PTSD was associated with a two-fold increase in risk of having comorbid ADHD (9). A recent genome-wide association study of 11 major psychiatric disorders, including ADHD, autism spectrum disorder, schizophrenia, mood disorders, and obsessive–compulsive disorder, showed that ADHD and PTSD were the most closely genetically correlated (13). Therefore, it has been suggested that ADHD and PTSD share a common neurobiological pathology (9). While patients with PTSD and ADHD are more difficult to treat, more likely to drop out of treatment, and tend to exhibit severe cognitive dysfunction, it was shown that the ADHD drug, atomoxetine (ATX), is effective in patients diagnosed with both disorders (10).

To our knowledge, there are no previous reports on comorbidity of chronic pain with PTSD and ADHD, therapeutic potential of ATX in improving cases of chronic pain with coexisting PTSD, and evaluation of cerebral blood flow in conjunction with the course of treatment for chronic pain with ATX. However, if chronic pain with PTSD is associated with ADHD, and if that pain and brain function can be improved with ADHD medications, it could provide an entirely novel solution against pain, thus, helping patients who are refractory to treatment. In this study, we present a case, in which ATX improved chronic pain with PTSD and improved cerebral blood flow.

## 2. Case description

The patient was a 56-year-old woman (height, 157 cm; weight, 45 kg); she had no childhood experiences of abuse or trauma and was an active girl with many friends. She graduated from high school and worked in an office until she married at the age of 22 years. No relevant medical history was reported in her family. She had a caring personality and took pleasure in helping others. She was in an adulterous relationship with her common-law husband, Mr. A, since 1995; she divorced her legal husband in 1998 because of being a victim of domestic violence (DV) and his relationships with other women.

The patient’s chief complaints were facial pain, headache between the eyebrows, and pain in the right upper extremity, which occurred at the site of another DV beating by Mr. A over a 6-year period from 1996 to 2002. Mr. A’s DV was severe, and approximately every 3 days he would repeatedly hit the patient all over her body with an iron pipe or strike her in the face with his fists. The patient could not leave him because of her responsibilities for debts of the company she ran with Mr. A. In January 2002, Mr. A was arrested and sentenced to 10 years in prison for repeated and prolonged sexual assaults on the patient’s daughter (who lived with them), and for confinement and attempting poisoning of the patient’s mother. On March 7, 2002, the patient’s mother was admitted to another hospital for treatment. The patient and her daughter were taken into custody and visited our hospital for physical and mental medical care.

### 2.1. Diagnostic assessments

This study was conducted in accordance with the World Medical Association’s Declaration of Helsinki and was approved by the Research Ethics Committee of Tokyo University Hospital (approval no. 3678). Written informed consent was obtained from the individual for the publication of any potentially identifiable data included in this article.

The patient was observed by a general internal medicine specialist, neurosurgeon, ophthalmologist, orthopedist, and psychosomatic physician. She was diagnosed with generalized bruising, left periretinal degeneration due to eye contusion, PTSD, psychosomatic headache, and insomnia; weekly counseling and regular psychosomatic treatment were prescribed. She received treatment at our hospital for 7 years. However, the pain in her face and right upper extremity persisted with no identifiable root cause. Hence, she was referred to our pain clinic in 2009. She was treated with trigger point injections and amitriptyline with no signs of improvement. For a prolonged period, she experienced catastrophic thoughts of being unable to bear the pain when she went out, supplemented with flashbacks and fears of her husband tracking her when he would be released from prison after serving his sentence. Hence, she was unemployed, stayed at home, receiving welfare benefits. Her 7-year treatment in our clinic included pregabalin, nortriptyline, paroxetine, fluvoxamine, sertraline, milnacipran, sulpiride, quetiapine, perospirone, chlorpromazine, and levomepromazine administration, but did not exhibit any improvement. Therefore, she was referred to SK, a psychiatrist in the pain clinic on April 27, 2016.

Patient visited the pain clinic once a month, completed the questionnaires described below each time, and received feedback from SK on the results. Subjective pain intensity was assessed using the numerical rating scale (NRS) (14). For the minimum clinically important differences (MCID), a decrease of ≥2 points in the NRS was considered either substantial or optimal (15). Health-related aspects of quality of life (QoL) were evaluated using the EuroQoL-5 Dimension (EQ-5D) (16, 17); scores of 0, 1.0, and 0.08 points indicated death, perfect health, and MCID, respectively (18). Anxiety and depression symptoms were assessed using the Hospital Anxiety and Depression Scale-Anxiety/Depression (HADS-A/D) (19); a score of ≥11 points was considered clinical level (20), and MCID on the HADS was 1.5 points (21). Pain-related catastrophizing thoughts were assessed using the Pain Catastrophizing Scale (PCS) (22); a score of ≥30 points represented the 75th percentile or higher in distribution of scores in chronic pain patients, with an MCID of 6.48 points (18). Each symptom of PTSD (Intrusion, Avoidance, Hyperarousal) was assessed using the Impact of Event Scale-Revised (IES-R) (23) and severity was assessed using the Post-traumatic Diagnostic Scale (PDS) (24). For the IES-R, a total score of ≥25 points was considered as positive PTSD screening score, and for the PDS, a score of ≥36 points indicated severe PTSD.

**Figure 1:**
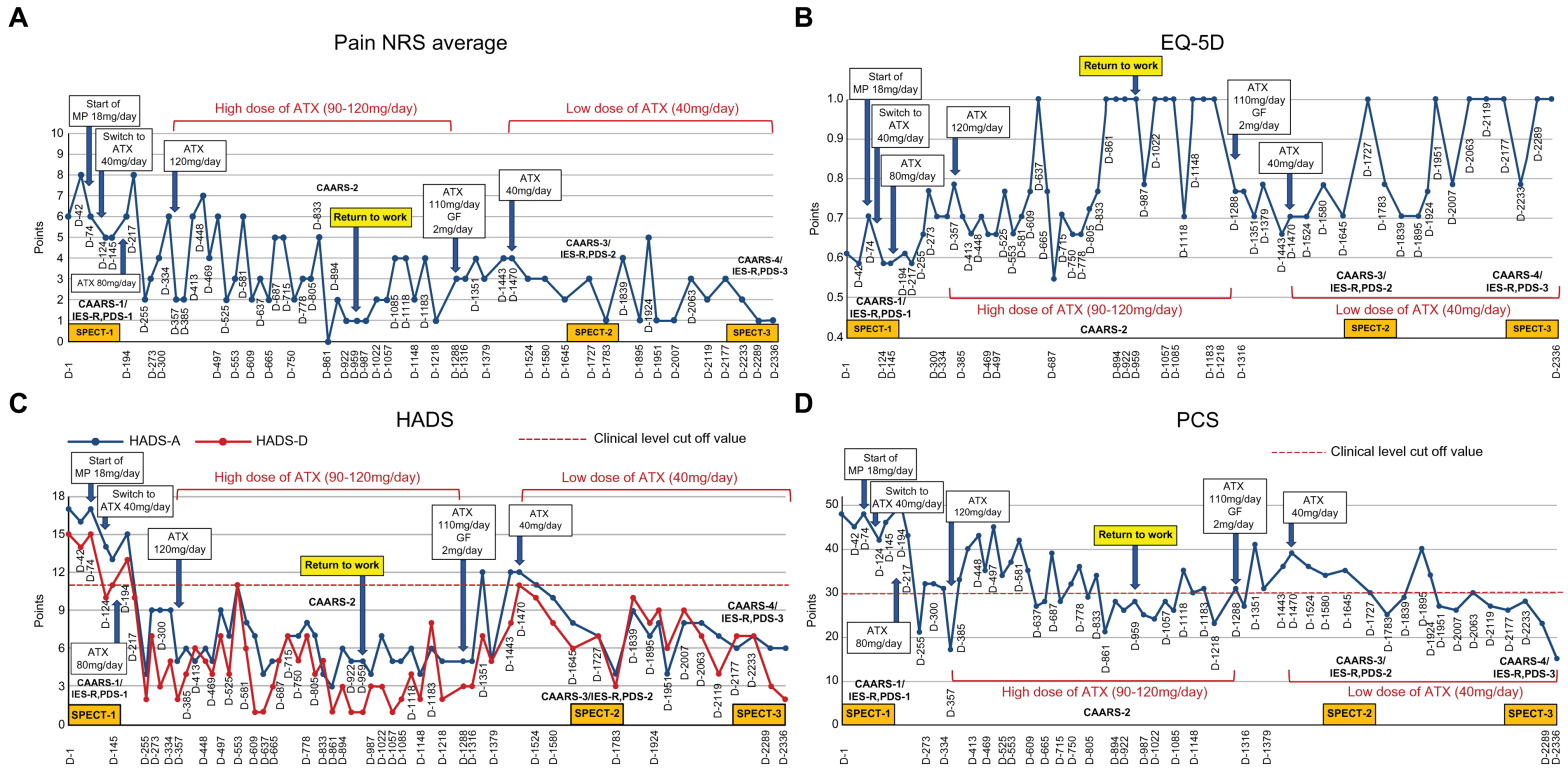
Course of treatment and objective/subjective parameters. **(A)** Pain NRS average; **(B)** EQ-5D; **(C)** HADS; and **(D)** PCS. ATX, atomoxetine; D, day; EQ-5D, euro QoL 5 Dimension; GF, guanfacine; HADS-A/D, Hospital Anxiety and Depression Scale-Anxiety/Depression; IES-R, Impact of Event Scale-Revised; MP, methylphenidate; NRS, numerical rating scale; PCS, Pain Catastrophizing Scale; PDS, Posttraumatic Diagnostic Scale; SPECT, single-photon emission computed tomography.

The patient’s average NRS for facial pain, headache, and right upper extremity pain at the first visit was 6/10, with an EQ-5D score of 0.612, HADS-A score of 17/21, HADS-D score of 15/21, PCS score of 48/52, and IES-R and PDS scores of 68 and 43 points, respectively (Figures 1 2).

**Figure 2:**
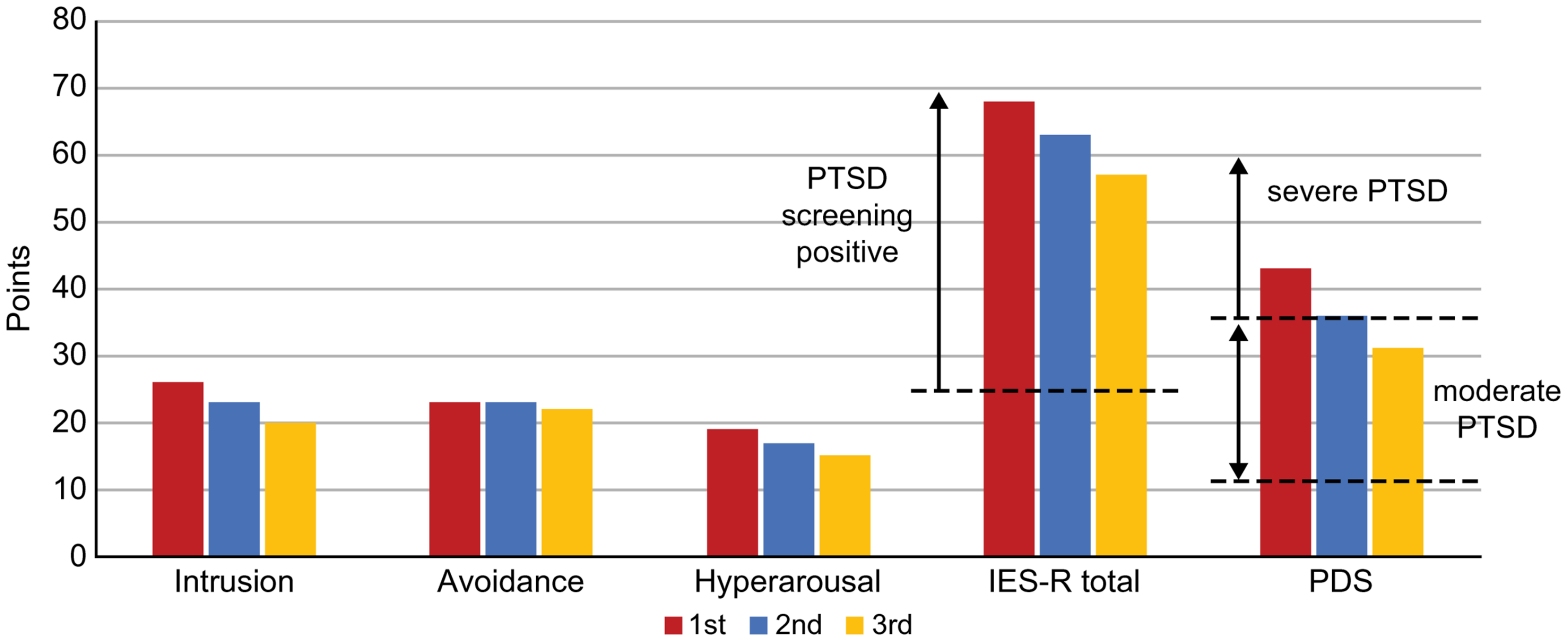
Changes in the IES-R and PDS scores along the course of treatment. IES-R, Impact of Event Scale-Revised; PDS, Posttraumatic Diagnostic Scale, PTSD, post-traumatic stress disorder.

A structured interview-the Mini-International Neuropsychiatric Interview (25), was conducted to differentiate between comorbid psychiatric disorders. She did not experience a major depressive episode with melancholy-type features such as hypochondriac delusions, manic episodes, obsessive–compulsive disorder, or psychotic disorder. Based on the DSM-5 (26) criteria, the patient was diagnosed with somatic symptom disorder (pain as the primary symptom).

**Figure 3:**
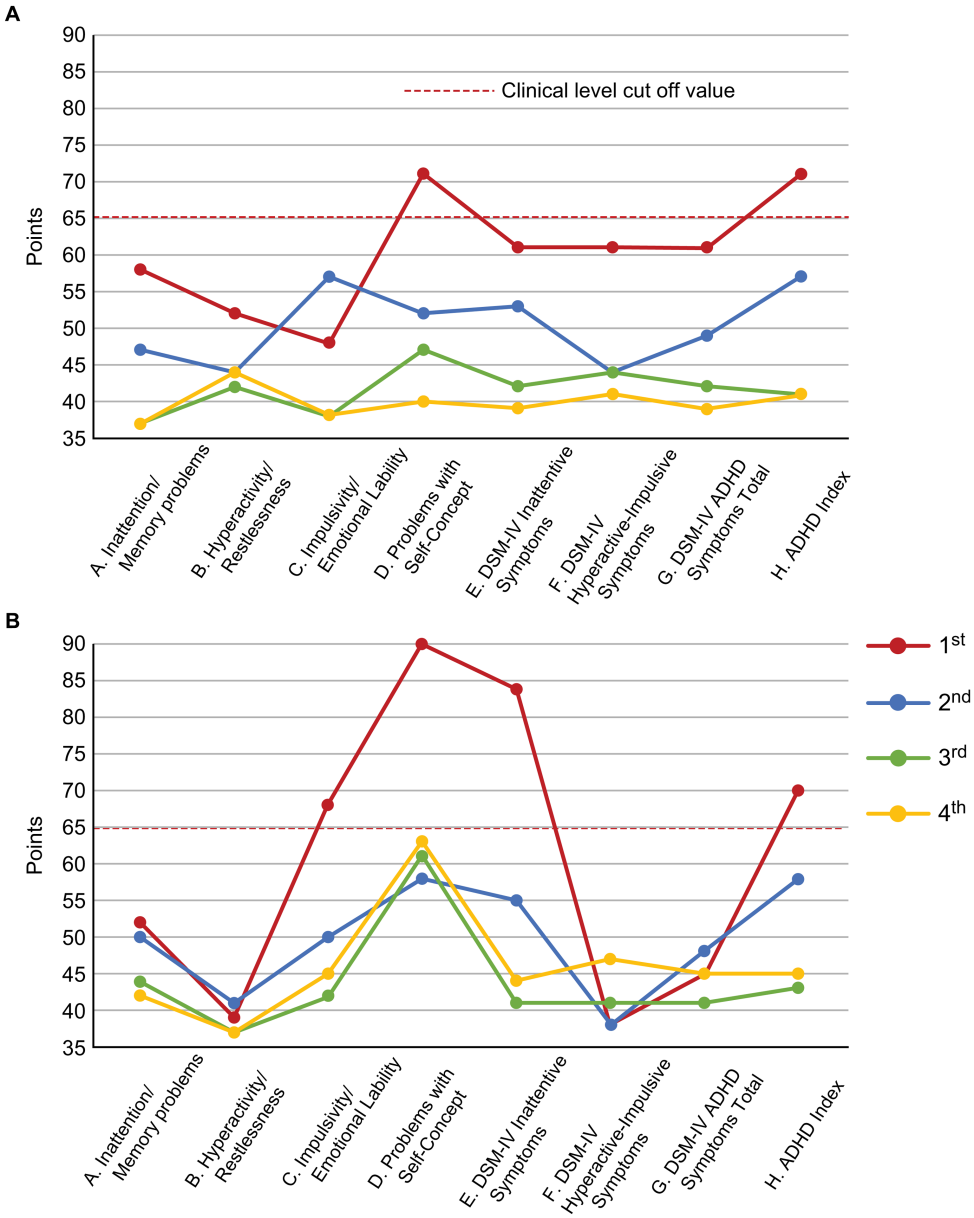
Changes in the CAARS scores along the course of treatment. **(A)** CAARS-S; **(B)** CAARS-O. ADHD, Attention-Deficit/Hyperactivity Disorder; CAARS-S/O, Conners’ Adult ADHD Rating Scale Self-report/Observer rated; and DSM-IV, Diagnostic and Statistical Manual of Mental Disorders, fourth edition.

During the examination, the patient talked a lot. However, her attention was easily diverted, topic was easily sidetracked, and consultation exceeded the scheduled time. After the consultation, she left her insurance card and prescriptions behind. Additionally, she used to fiddle with her hair and fidget with her hands as she spoke. As ADHD has been reported to coexist frequently with chronic pain (2), SK suspected ADHD co-morbidity in this patient, and conducted ADHD symptom assessment and diagnosis confirmation from Day 1 to Day 74. ADHD symptoms were assessed using the Conners’ Adult ADHD Rating Scale (CAARS); self-report (CAARS-S) and observer forms (CAARS-O) (27) were completed by the patient and her daughter, respectively. According to the CAARS results, her subscale scores exceeded by 65 points and her ADHD symptoms were assessed as being at the clinical psychiatric level (Figure 3). Since childhood, she had been easily distracted, had difficulty in reading, and was the most forgetful in class. She was often bullied for forgetting and failing to follow rules when playing with friends. She was diagnosed with combined type ADHD and she satisfied six out of nine DSM-5 ADHD diagnostic criteria for inattention and five out of nine criteria for hyperactivity-impulsivity.

### 2.2. Therapeutic interventions and outcomes

**Figure 4:**
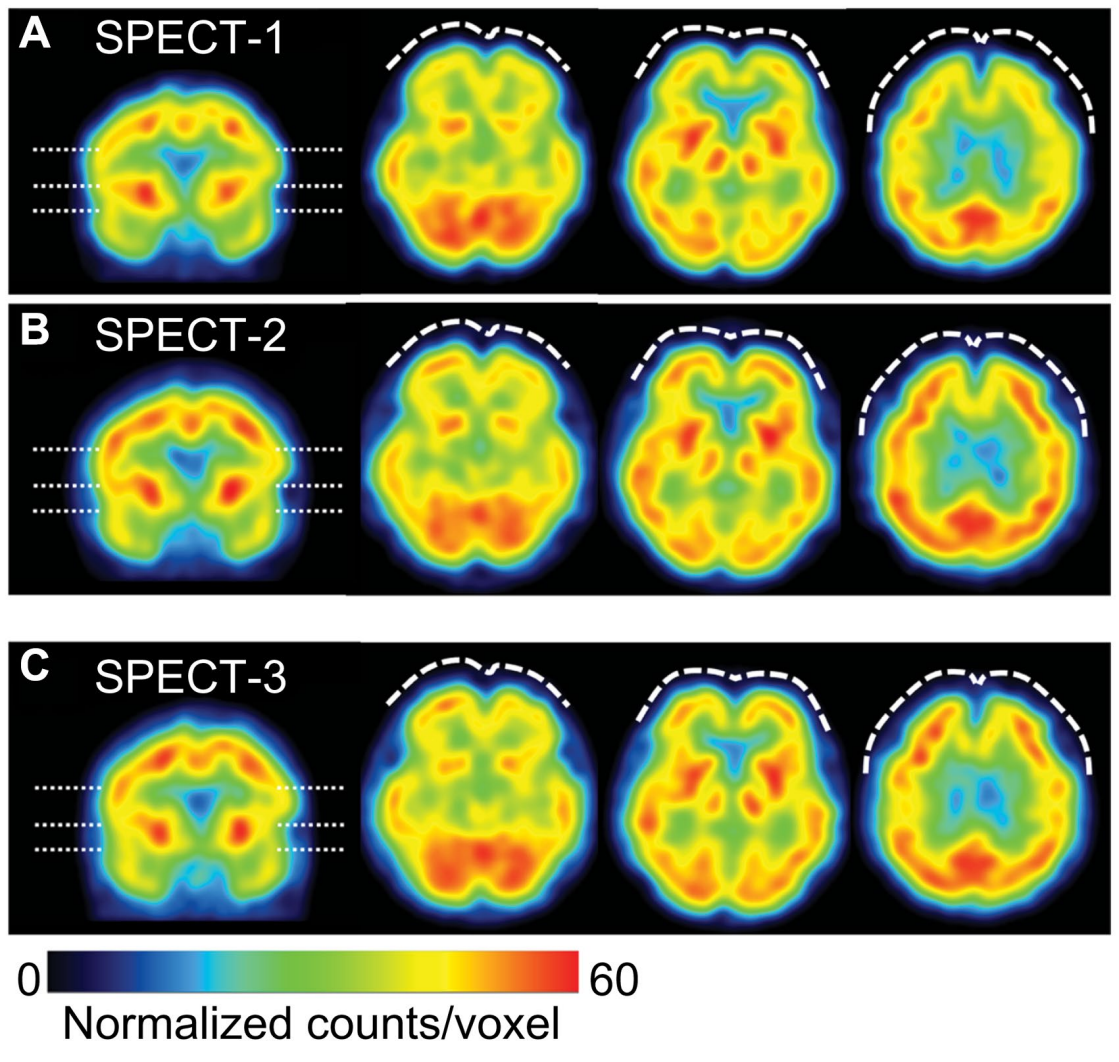
Representative images of cerebral blood flow (CBF) SPECT. The voxel values are normalized by the average counts-per-voxel in the cerebellum being 50, then the color ranges from 0 to 60. The left image is a coronal view, indicating the slice levels of the right three axial images by dashed lines. CBF-SPECT on day 74 before treatment **(A)** shows bilateral frontal hypoperfusion (dashed curves), which is improved on day 1727 **(B)** and on day 2289 **(C)** during low-dose atomoxetine administration. SPECT, single-photon emission computed tomography.

On Day 74, single-photon emission computed tomography (SPECT) was performed prior to treatment initiation. 99mTc-ethyl cysteinate dimer (ECD; Fujifilm RI Pharma, Tokyo, Japan) at a dose of 740 MBq was administered to the patient resting in a supine position in a quiet room with her eyes closed. At 10 min post-injection, SPECT was performed for 30 min with a triple-head gamma camera (GCA-9300R; Canon Medical Systems Corp, Tochigi, Japan). The results showed hypoperfusion in the bilateral prefrontal and temporal cortex (Figure 4).

On Day 74, administration of methylphenidate 18 mg/day was discontinued owing to adverse side effects. Day 96 involved switching from methylphenidate to ATX (40 mg/day). On Day 124, she could go out only when invited by friends. On Day 145, ATX increased to 60 mg/day, rendering her able to tidy the house for the first time in several years. On Day 166, ATX further increased (80 mg/day). On Day 245, she started socializing more and called her friends for the first time in 15 years and chatted happily, researched qualification exams, and searched for part-time work. She stated that she cried after watching TV dramas and revealed the return of real emotions. Her ATX dose increased to 90 mg/day, while on Day 273, the dose further increased (100 mg/day) as she stated that although her brother was hospitalized in critical condition, she no longer felt so depressed and could go visit him even though her face hurt. On Days 329 and 357, ATX further increased to 110 mg/day and 120 mg/day, respectively. She stated that she could move around despite the pain, concentrate on reading, and perform things as planned. Thereafter, ATX was maintained at a high dose (approximately 90–120 mg/day) until Day 1,288, with gradual improvement in each pain-related scale score reported.

On Day 894, the NRS, EQ-5D, HADS and PCS scores showed clinically significant improvements beyond MCID. The CAARS was performed for a second time, and we found that the scores on all subscales had improved to the normal range. On Day 959 she could return to work for the first time in 11 years. However, during the period of high ATX doses (Day 329–Day 1,288), she often took ATX intermittently due to experiencing side effects of nausea, weight loss, and hyperhidrosis. When she stopped taking ATX, her conversations during consultations were again derailed and diffused. Results of each pain-related scale worsened, and the progress graphs often showed large, repeated waves of fluctuation.

On Day 1,288, she wanted to switch medicines due to ATX painful nausea side effects. Hence, 2 mg/day of guanfacine was added to 110 mg/day of ATX. However, the experience of various side effects, including low blood pressure, dizziness, and weight gain, resulted in the self-discontinuation of ATX with guanfacine. Thereafter, each pain-related scale score worsened. When ATX was resumed at 40 mg/day on Day 1,470, each scale’s scores were improved. ATX administration was maintained at a low dose of 40 mg/day from Day 1,470 to Day 2,336, which resulted in sustained improvement in pain-related symptoms. On Day 1727, the CAARS was performed a third time and the IES-R, PDS, and SPECT were performed for a second time; on Day 2,289, the results of fourth CAARS and third IES-R, PDS, and SPECT showed improvement from the previous ones.

### 2.3. Patient perspective

She stated that she had felt a strong sense of guilt regarding receiving unemployment benefits for many years. Now she could work, contribute to society, and regain her self-confidence. She also expressed gratitude, saying, “The diagnosis of ADHD in the course of pain treatment was unexpected but very helpful in getting to know myself better.”

## 3. Discussion

To the best of our knowledge, this is the first reported case of ADHD coexisting with chronic pain accompanied by PTSD. It is reported that in psychiatric practices, the diagnosis of more than 80% of adult ADHD cases is missed (28). As cases of chronic pain with PTSD are often assigned to pain clinicians or orthopedic surgeons who are unfamiliar with ADHD treatment, ADHD comorbid with chronic pain is more likely to be overlooked. Hence, in cases of chronic pain with comorbid PTSD, aggressive screening for ADHD should be considered. In the present case, ADHD comorbidity was highly suspected by screening using CAARS, although there was no previous diagnosis of ADHD.

The patient’s tendency to be “caring and over-indulgent” corresponds to the hyperactivity and impulsivity component of ADHD, and her “inability to organize her thoughts quickly and cause an argument to defend herself against an attack” corresponds to the inattentiveness symptoms of ADHD. These symptoms of ADHD accompanied by low self-esteem (CAARS-S/O subscale D. Problems with Self-Concept) were presumed to have triggered DV both from her legal husband and Mr. A. It is known that at the site of a DV-related injury, excessive muscle tension can occur in anticipation to reduce the impact of the next violence, resulting in pain (29). In this case, the patient developed chronic pain in the face, head, and right upper limb, consistent with the DV injury sites.

In this case, ATX, an ADHD treatment, improved chronic pain with PTSD. The relationship between prefrontal cortex performance and noradrenaline (NA) and dopamine (DA) levels follows an “Inverted U-shaped” function. Maximal performance is achieved when both neurotransmitters are in moderation (30). ATX inhibits NA transporters in the prefrontal cortex and moderates NA and DA transmission, thereby enhancing the prefrontal cortex performance and improving ADHD symptoms (30). Furthermore, the prefrontal cortex is also functionally coupled with the descending pain inhibitory system, acting as a virtual filter to reduce unpleasant sensations like pain and itching (31, 32). Therefore, ATX has been reported to improve chronic pain associated with ADHD (7, 33).

Exposure to intense chronic stress, such as PTSD, causes phasic over-firing of NA and DA in the prefrontal cortex, exacerbating anxiety, and inattention due to hyperarousal. However, “long-term treatment” with ATX restores tonic neurotransmission of NA and DA in the prefrontal cortex, down-regulates phasic over-transmission of NA and DA, and desensitizes postsynaptic NA and DA receptors. As a result, the prefrontal cortex performance is increased, hyperarousal-generated anxiety is reduced, and ADHD-related symptoms are improved (30). In addition to improvements in CAARS, in our case, the ATX also improved scores on the IES-R sub-scales “Intrusion,” reflecting excessive attention to repetitive and intrusive trauma-related thoughts, and “Hyperarousal,” reflecting over-arousal. Our findings suggest that the improvement of chronic pain with PTSD with ATX is consistent with the theoretical pharmacological effects of ATX as described above.

The improvement in pain scores reported by the NRS and HADS was associated with higher ATX doses (90–120 mg/day). However, ATX administration was intermittent because of side effects and fluctuations recorded on each scale. When ATX was re-entered at a lower dose of 40 mg/day from Day 1,470 to Day 2,336, the degree of improvement was more modest than that at higher doses. Treatment was not interrupted and was maintained for longer periods, which resulted in improvements in pain-related measures, ADHD symptoms, and PTSD symptoms. The recommended ATX dose for adults is 80–120 mg/day (34), but when significant side effects emerge, a lower dose (e.g., 40 mg/day) with emphasis on “long-term maintenance” may lead to more stable improvements.

In the present case, along with the improvement in chronic pain with PTSD by ATX, there was also an improvement in cerebral blood flow, which, to our knowledge, is an insight that is firstly described. Functional brain imaging studies of patients with PTSD have reported hypo-reactivity of the medial prefrontal cortex, hyper-reactivity of the amygdala, and abnormal hippocampal responses (35). As impaired prefrontal cortex performance in patients with PTSD is regulated via NA and DA balance, it was thought that ATX may be successful in treating chronic pain associated with PTSD. Evidence for this was observed in the improvement of blood flow in the prefrontal cortex in three SPECT sessions conducted in our case, following ATX treatment. Previous studies using SPECT of ATX have reported that ATX reduced the bioavailability of DAT in the striatum in adolescents with ADHD (36) and increased cortical blood flow in the affected area in aphasia after stroke (37). In the present case, SPECT showed blood flow improvement in both striatum and cortex, which was consistent with previous studies. Changes in SPECT images observed in parallel with the course of treatment in the present case suggest that SPECT could be an objective measure of the effectiveness of chronic pain treatment (38).

There are three limitations to this study. First, the improvement in cerebral blood flow in this case may not only reflect the effect of ATX, but also be attributed to the patient’s resumption of work and reintegration into society. Second, as the course of treatment in this case was 6.5 years, the idea that the long passage of time since the onset of DV may have naturally reduced PTSD symptoms, which in turn may have improved pain, ADHD symptoms, and cerebral blood flow, should not be fully ruled out. Third, prolonged exposure, cognitive processing therapy, eye movement desensitization, and reprocessing therapy are usually recommended as treatments for PTSD (39). However, it has been noted that patients with PTSD tend to be less focused to receive these therapies in the first place, are less likely to benefit from them, and are more likely to drop out of treatment (9, 10). Moreover, because this tendency is more pronounced in cases of PTSD with ADHD, it has been suggested that ADHD medications should be used prior to receiving PTSD-specific treatment. In addition, as comorbid PTSD and ADHD can mutually exacerbate each other’s symptoms, it can be expected that medication for ADHD will also reduce PTSD symptoms (10, 40). Furthermore, it has been noted that there is a lack of psychologists in chronic pain healthcare settings, such as outpatient pain clinics, who are able to provide specialized treatment for PTSD (41). Therefore, medication for cases of PTSD with comorbid ADHD, as that presented in this report, could be an important treatment approach.

## 4. Conclusion

This study showed that chronic pain with PTSD may be comorbid with ADHD, and that ATX can improve cerebral blood flow and chronic pain associated with PTSD. Although the diagnosis of ADHD comorbid with chronic pain is easily missed, aggressive screening is important because once the diagnosis of comorbidity is confirmed, the option of an ADHD treatment, such as ATX, can be selected. Based on our results, ATX may be an ideal candidate for treatment for chronic pain after PTSD with ADHD. However, to our knowledge, this is the only case report reporting an association between chronic pain after PTSD and ADHD. Further research is needed to establish the inter-relationship between these disorders and their treatment.

## Data availability statement

The original contributions presented in the study are included in the article, further inquiries can be directed to the corresponding author.

## Ethics statement

This study was approved by the Research Ethics Committee of Tokyo University Hospital (approval no. 3678). Written informed consent was obtained from the patient for the publication of this case report.

## Author contributions

SK, S-IN, and KM conceived the work and interpreted the data. SK, MT, and NS collected the data. SK, MT, and ToM managed the data. SK, MT, and TaM drafted the manuscript. SK, TaM, S-IN, and KU prepared the final manuscript. All authors approved the final manuscript.

## Funding

This study was supported by a Grant-in-Aid for Scientific Research (C) from the Japan Society for the Promotion of Science (grant number JP20K07755).

## Conflict of interest

The authors declare that the research was conducted in the absence of any commercial or financial relationships that could be construed as a potential conflict of interest.

## Publisher’s note

All claims expressed in this article are solely those of the authors and do not necessarily represent those of their affiliated organizations, or those of the publisher, the editors and the reviewers. Any product that may be evaluated in this article, or claim that may be made by its manufacturer, is not guaranteed or endorsed by the publisher.
